# β-1,3-glucan improved the health and immunity of juvenile African catfish (*Clarias gariepinus*) and neutralized the histological changes caused by lead and fipronil pollutants

**DOI:** 10.1186/s12917-023-03585-5

**Published:** 2023-02-11

**Authors:** Gamal A. Elmowalid, Wael A. M. Ghonimi, Hossam M. Abd Allah, Haytham Abdallah, Abdelhakeem El-Murr, Ashraf M. Abdelwahab

**Affiliations:** 1 Department of Microbiology, Faculty of Veterinary Medicine, Zagazig University, Zagazig, Egypt; 2 Department of Histology and Cytology, Faculty of Veterinary Medicine, Zagazig University, Zagazig, Egypt; 3 Department of Biochemistry, Faculty of Veterinary Medicine, Zagazig University, Zagazig, Egypt; 4 Department of Fish Diseases and Management, Faculty of Veterinary Medicine, Zagazig University, Zagazig, Egypt

**Keywords:** β-1,3-glucan, Catfish, Fibronil and lead toxicity, Fish cytokines, Histopathology

## Abstract

**Background:**

Water pollutants cause adverse effects in aquatic ecosystems. The immunomodulatory and mitigating effects of dietary 1,3-glucan on fipronil and lead-induced intoxication in African catfish (*Clarias gariepinus*) were investigated. Two hundred forty catfish were randomly divided into four equal groups: those in the first group were fed basic diet and served as controls; those in the second group were supplemented with β-1,3-glucan (0.1%); those in the third group were exposed to combination of lead nitrate at 0.041 mg/L (1/10 96 h LC50) and fipronil at 2.8 mg/l (1/10 96 h LC50); and those in the fourth group were exposed to combination of fipronil, lead, and β-1,3-glucan. The health status, haematological, immunological, and histological changes were all evaluated.

**Result:**

Swelling on the dorsolateral side, spinal column deviation, sluggish movement, skin bleaching, excessive mucus secretion, significant variations in blood indices-related measures, and a 45% death rate were observed in the third group. There was a significant reduction in interleukin-1 (IL-1) and interleukin-6 (IL-6) and immunoglobulin M (IgM) concentrations, as well as decrease in their corresponding gene expression, indicating that fipronil and lead had immunosuppressive activity. Severe catarrhal enteritis and mucinous degeneration of the lining epithelium, and notable depletion of white pulp, congested red pulp and hemosiderosis were common pathological findings in the spleen. β-1,3-glucan alone or in combination with fipronil and lead provoked physical activity, blood indices, with elevations in IL-1β, IL-2, IL-6, and IgM concentrations, as well as up-regulation in their genes’ expression in splenic tissues, when compared to the third group. The spleen and intestine had normal histological architecture with 5% mortalities. There were no fish deaths in the β-1,3-glucan-alone or control groups.

**Conclusion:**

The use of β-1,3-glucan (0.1%) as dietary supplement could be implemented to protect against the toxic effects of fipronil and lead toxicity by improving the health and immunological parameters of intoxicated catfish.

**Supplementary Information:**

The online version contains supplementary material available at 10.1186/s12917-023-03585-5.

## Introduction

Aquaculture is a thriving industry aimed at meeting the expanding edible fish market. In developing countries, fish meat is a good source of protein from both a practical and economic standpoint [1]. The ecosystem and food additives used in aquaculture systems influence the quality and safety of fish and fish meat. Water pollutants, climate change, nutrient shifts, acidification, habitat loss, exploitation, biological invasions, and chemical contamination are just a few of the stressors that threaten freshwater ecosystems and the fish industry [2]. Contamination of water with large amounts of pesticides has been shown to affect fish and other organisms in aquatic ecosystems’ growth rates and reproduction with evidence of tissue damage. Insecticides are transported into urban streams and waterways via irrigation runoff and storm water, even if they are not applied near surface water bodies, provoking adverse effects not only on the target species but also on a wide range of non-target organisms that inhabit aquatic ecosystems, such as invertebrates, birds, and fish [3–10]. Impairment of fish immune function as a consequence of polluted aquatic environments can result in diseases susceptibility. The fish’s “innate and adaptive immune responses” play a pivotal role in their protection against microbial infections [11]. Cytokines and immunoglobulins are involved in fish various biological activities and are responsible for the signaling cascades required for the promotion of inflammatory reactions and the modulation of both innate and adaptive immune responses. They are also essential in regulating the immune system to mount the proper adaptive immune response to successfully remove pathogens and control infection, promote immune cell proliferation, and repair damaged tissue caused by inflammation [12, 13]. IgM elicits effective, specific humoral responses against various microbes and protects against recurrent infections [14]. Changes in cytokines, immunoglobulins, and other mediator levels were all used as indicators to assess the direct effect of pesticide exposure [15].

Fipronil is a new, fast-growing, broad-spectrum N-phenylpyrazole insecticide that is used extensively in global markets, public health, and agriculture because of its efficacy at low doses [16], despite its adverse health and environmental effects. Exposure to fipronil is linked to negative health outcomes in humans and animals, including promoting neuronal cell injury, which results in apoptosis through the production of reactive oxygen species (ROS) [17]. Its mechanism of action is to inhibit excitable membranes by targeting the γ-aminobutyric acid (GABA) receptor chloride complex. Fipronil’s blockage of GABA-gated chloride channels reduces neuronal inhibition, resulting in hyper-excitation of the target host’s central nervous system, convulsions, and, eventually, death [18, 19]. Several reports of Adverse Drug Experiences (ADEs) associated with the use of fipronil-containing products have been reported in target and non-target animal species (honeybees, fish, aquatic invertebrates, and upland game birds), as well as in humans either applying the product or handling the target animal after application. According to the United States Environmental Protection Agency (US EPA), fipronil is highly toxic to fish, such as bluegill sunfish (LC 50 = 0.083 ppm) and rainbow trout (LC 50 = 0.246 ppm). Many fish species are very sensitive to the effects of fipronil [16–19]. Fipronil has been shown in studies to have sub-lethal effects ranging from genotoxic and cytotoxic effects and impaired immune function to reduced growth at concentrations below those associated with mortality. The toxicity varies depending on the fish species and exposure route. The gills of Caspian white fish are affected by fipronil waterborne [20], as are behavioral and skin colour changes in juvenile brown shrimp [21]. Furthermore, changes in the behavioral and physiological processes of *Daphnia magna* have been [22]. Moreover, fipronil has been reported to be very toxic to Nile tilapia, where it can accumulate in the tissue and lead to toxicity to cells and genes and mortality [23, 24].

It also caused severe toxicity in zebrafish embryos by disrupting higher-level genomic DNA methylation, which was involved in cell signaling and development [25]. Furthermore, fipronil metabolites, such as sulfone and sulphide, are more toxic than fipronil itself [26], Also, it was reported that fipronil is highly toxic to *Aristichthys nobilis* fish [27].

Because of its low rate of elimination, lead (Pb) is one of the most dangerous pollutants in the environment that can accumulate in the body [28]. It enters aquatic systems through urban, mining, and agricultural runoff, atmospheric precipitation, lead-containing fertilizers and gasoline, and a variety of other routes [29, 30]. Lead has been shown in studies to cause neurological, haematological, gastrointestinal, reproductive, circulatory, immunological, histopathological, and histochemical changes, as well as a variety of other undesirable effects in a dose and time-dependent manner [31–33]. Lead acetate had Immunotoxicologic effects and damage of intestinal epithelium of freshwater fish *Channa punctatus*. It interfered with bacterial phagocytosis, intracellular killing capacity, cytokines production, and cell adhesion as well as inhibited release of antimicrobial substances like nitric oxide and myeloperoxidase by intestinal macrophages [34]. Pollutants not only affect aquaculture, but also indirectly affect human. It has been found that some pollutants can transfer to the fish meat consumers. Pollutant tissue residues in fish meat and consumption to these residues affects humans health [16]. Environmental pollution is one of the problems that current aquaculture suffers from. Repeated exposure to sub-lethal concentrations of pesticide pollutants has been associated with physiological and behavioral changes that reduce fish populations, decrease immunity to diseases, and decrease predator avoidance [35].

The search for natural substances used as dietary feed supplements is to improve fish health and immunity status, which are required to prevent infection and the transfer of pollutant residues to consumers. Prebiotics have recently gained attention in aquaculture for disease control and competition with various environmental stressors, as well as to promote the growth of cultured fish. β-glucans are used prebiotic in aquaculture, and it has been widely used to reduce the negative effects of stress and improve various physiological parameters [36]. β-glucans administration increased food conversion rates in *Labeorohita* fingerlings growth [37], *Pagrus auratus* snapper growth [38] and improved rainbow trout survival and resistance to infection [36]. β-glucan was discovered to increase the production of crap antibodies [39, 40] post *Aeromonas hydrophila* challenge. Food supplementation with β-glucan improved ammonia-related stress in *Oreochromis mossambicus* by improving cellular, humoral, and antioxidant responses [41]. Rainbow trout’s complement fraction 3 (C3) gene expression was altered, and inflammatory cytokines gene expression was up-regulated after -glucan bath immunostimulation [27].

From the aforementioned, it is documented that fipronil insecticide or lead heavy metal entering waterways can act as stressors, disrupting the ecosystem and resulting in impairment of physiological parameters, growth retardation, immunosuppression, and increased mortality in fish. African catfish, *Clarias gariepinus*, a commonly consumed tropical freshwater fish in many countries and is one of the most cultured fish both inside and outside its natural range of tropical and subtropical environments [42]. They are also one of the most cultured fishes in the world and can be affected by pesticide administration [43]. So, it was of paramount importance to develop a strategy to reduce the side effects of those pollutants in catfish culture and to protect humans from the tissue residues of those pollutants. In this scenario, we hypothesize that adding immunomodulatory agents such as β-1,3-glucan prebiotic to the diet could neutralize lead and fipronil stressors’ activities, strengthen catfish vital defense elements, inhibit pathological changes, and improve health status. As a result, the aim of this study was to explore how β-1,3-glucan supplementation affected the immunomodulatory activities and health status of catfish in response to induced fipronil and lead intoxication. Blood parameters, cytokines’ gene expression (IL-2 and IL-6), and cytokines’ (IL-1β, IL-2 and IL-6) and IgM serum concentration were all used to assess the immunomodulatory status, in addition to physiological activities and pathological changes in the spleen and intestine, to evaluate the health status. This would have been a cost-effective way to improve fish health and protect fish consumers from the tissue residues of those pollutants.

## Materials and methods

### Chemicals

#### Fipronil

Fipronil 20% was obtained from YongNong Biosciences Co., Ltd. China. Technical grade Fipronil (C12H4Cl2F6N4OS) (99.1% pure) manufactured by Bio Quest International Private Limited, Mumbai, India and it was consisting of γ and β isomers at the ratio of 50:50. Fipronil doses were estimated [23].

#### Lead

The solid white crystal form of lead nitrate Pb(NO3)2 was obtained from Fisher Scientific Company in Canada.

#### Beta glucan

β-1, 3-glucan (Sigma, U.S.A.) was dissolved in phosphate buffered saline (PBS), thoroughly mixed and subsequently added to basic diet constituents at a rate of 0.1% before palletization. The feeding period and β-1, 3 glucan dose used in this study were based on the earlier report [43].

#### Cytokines and antibodies

Fish Interleukin-1β (IL- 1β), ELISA Kit, Cat. No. MBS700023 and Fish Interleukin 6, (IL-6) ELISA Kit, Cat. No. MBS015740 were used in this study for detection of IL-1β and IL-6 levels. The kits were obtained from MyBiosource Co. (San Diego, California, USA). Immunoglobulin M (IgM) ELISA Kit. Catalog No. CSB-E12045Fh was purchased from CUSABIO BIOTECH CO., Ltd. and it was used to quantitatively determine the IgM level.

### Fish management and maintenance

Two hundred forty Nile catfish with an initial 40 ± 3.0 g body weight (mean ± SD) and 14.71 ± 1.23 cm of fork length were used in this study, and they were obtained from a local hatchery (Abbasa, Ash Sharkyia, Egypt). The fish were transferred to the Department of Fish Diseases and Management, Faculty of Veterinary Medicine, Zagazig University, Egypt, where the experiments were performed. Initially, fish were immersed in a salt bath of NaCl at a concentration of 2.5% for 5 min to get rid of external parasites and fungal infections. The fish were acclimatized for 2 weeks before starting the actual experiments, which lasted a total of 2 months. During the acclimatization period, fish were fed a commercial pelleted feed containing 32% protein and were placed in glass aquaria (80 × 40 × 30 cm capacity). Each aquarium had 60 l of chlorine-free tap water. An aerator was used to provide air supply; a thermostatically controlled heater kept the water temperature at 26 °C; the pH of the water was kept at 6.5–7.0; dissolved oxygen averaged 60.5 mg L-1; ammonia averaged 0.01 mg L-1; and nitrite was 0.20 mg L-1. These parameters were measured routinely with a freshwater kit (La Motte®, Chertes twon, MD, USA). Approximately 30% of the water was changed daily, and a constant water flow was maintained.

### Experimental design

After the acclimatization period, the 240 catfish were divided into four experimental groups (60 fish per group, each group in triplicate) after acclimation, and then placed into 16 aquaria at random (4 aquaria per group with 15 fish per aquarium). The first group served as a control, and the fish were fed a basic diet without any additions or treatments, while the fish in the second group were fed a basic diet supplemented with 0.1% of β-1, 3-glucan, and fish in the third group were fed a basic diet and exposed to a combination of lead nitrate at 0.041 mg/L (1/10 96 h LC50) and fipronil at 2.8 mg/l (1/10 96 h LC50). The fourth group of fish received a basic diet supplemented with 0.1% of 1, 3 β-glucan before being exposed to lead nitrate and fipronil at the same mentioned concentrations. All of the fish in the various experimental groups were fed their individual diets ad libitum four times daily at a rate of 3% of their body weight. Every day, the fish were checked for any changes in their general health, clinical signs, odd behavior or coloration, or respiratory distress. The affected fish’s post-mortem lesions and mortality rate were documented.

### Immunological and biochemical parameters evaluation

At the end of the feeding experiment, ten randomly selected fish from each group were collected and sedated with tricaine methane sulfonate MS-222 (100 ppm) for caudal vein blood sampling. Each blood sample was divided into two aliquots. One aliquot was placed in EDTA-containing tubes and was utilized for haematological analysis [44], and the second aliquot was allowed to clot at 4°C and centrifuged at 1500 x g to separate the sera, which were then aliquoted and stored at -20°C for ELISA assay. IL-1 and IL-6 cytokines, as well as immunoglobulin (IgM) levels in the various test groups, were measured, as well as some biochemical parameters. Both ELISA and haematological assays were carried out as directed by the kits’ manufacturer.

### Tissue sampling

Intestinal and splenic tissues were obtained before and after the catfish treatments with the tested compounds in the four groups. Splenic tissues were employed for both histopathological and molecular detection of the target inflammatory cytokines’ gene expression. Intestinal tissues were used for histopathological investigations.

### Histopathological investigations

Specimens from the intestine and spleen were collected and immediately fixed in a 10% buffered neutral formalin solution for 48 hrs, then processed histologically, where the specimens were dehydrated in ascending grades of ethanol (70–100%), cleared in xylene, and finally embedded in paraffin. Five-micron thick paraffin sections were prepared and then routinely stained with Hematoxylin and Eosin (H&E) dyes for any histological changes according to [45]. The microphotographs were taken using a digital Dsc-W 130 super steady cyper shot camera (Sony, Japan) connected to an Olympus BX 21 light microscope.

### Molecular determination of IL-1β, IL-2, or IL-6

Spleens were obtained from dead or euthanized fish, quickly maintained in liquid nitrogen and stored at -80°C to be used in a semi-quantitative RT-PCR [46] using the appropriate specific set of primers to measure IL-1, IL-2, or IL-6 cytokine gene expression (Table 1). RNA was extracted from various fish groups using the Gene JET RNA purification kit (Fermentas, UK) according to the manufacturer’s instructions. A Nano-Drop®ND-1000 Spectrophotometer (Wilmington, Delaware USA) was used to verify the quantity and purity of the RNAs collected. The reverse transcriptase enzyme Superscript II RNase H (Invitrogen, Carlsbad, CA, USA) was used to make cDNA To express arbitrary units of relative abundance, the values of the specific targets were normalized to those of β-actin. After amplification, PCR products were run on a 2% agarose gel in 90 mM Trisborate, 2 mM EDTA buffer (TBE), pH 8, and stained with ethidium bromide and observed by UV transillumination. To check the size of amplification products, a DNA ladder molecular weight marker (Gel Pilot 100 bp ladder (Cat. No. 239035) supplied by (QIAGEN, USA) was employed. Consequently, the products densities were analyzed by gel documentation system (Bio Doc Analyze, Biometra, Germany) and photographs were taken using a Sony XC-75 CE camera (VilberLourant Inc. Cedex, France) with the density of the bands assessed using Photo-Capt v.99 Image software (VilberLourant Inc. Cedex, France).

**Table 1:** Primers nucleotide sequences (5′–3′) of IL-1β, IL-2, IL-6 and β-actin

Gene	Primers	Gene Bank ID	Product size	Reference
**IL-1β**	F: AGGTGACACTATAGAATAT GAACCTGTCGACCTACGTG R: GTACGACTCACTATAGGGA ATACCAGGGTGCAGAGGTTG	AB704199	271	Kono et al., 2002 [47]
**IL-2**	F: AGGTGACACTATAGAAT AG GA CGATCATCATTGCATCA R: GTACGACTCACTATAGGGA GGATTTCTCGATCTTCACGC	NM.001037994	200	Bird et al., 2005a [48]
**IL-6**	F: AGGTGACACTATAGAATA TGAATGGTGATTCAGACCCA R: GTACGACTCACTATAGGG A GAAAAAGCTGCTCGGCTCTA	NM.001032722	229	Bird et al., 2005b [49]
**β-actin**	F: AGGTGACACTATAGAATAC GTCATGGACTCTGGTGATG R: GTACGACTCACTATAGGGA G TCAGGCAGCTCGTAGCTCT	U37499	317	Venkatesh et al., 1996 [50]

### Statistical analysis

SPSS version 20 was used to perform statistical analysis on the data. Statistical packages (IBM 1, New Orchard Road, Armonk, New York 10,504–1722, United States) presented as a mean ± SD, *n* = 10 were used. Statistical differences among groups were performed using a one-way analysis of variance (ANOVA). Duncan’s test was used to test inter-group homogeneity. The statistical significance level was set at *p* ≤ 0.05.

## Results

### The mortality rate in different African catfish (*Clarias gariepinus*) groups

**Fig. 1:**
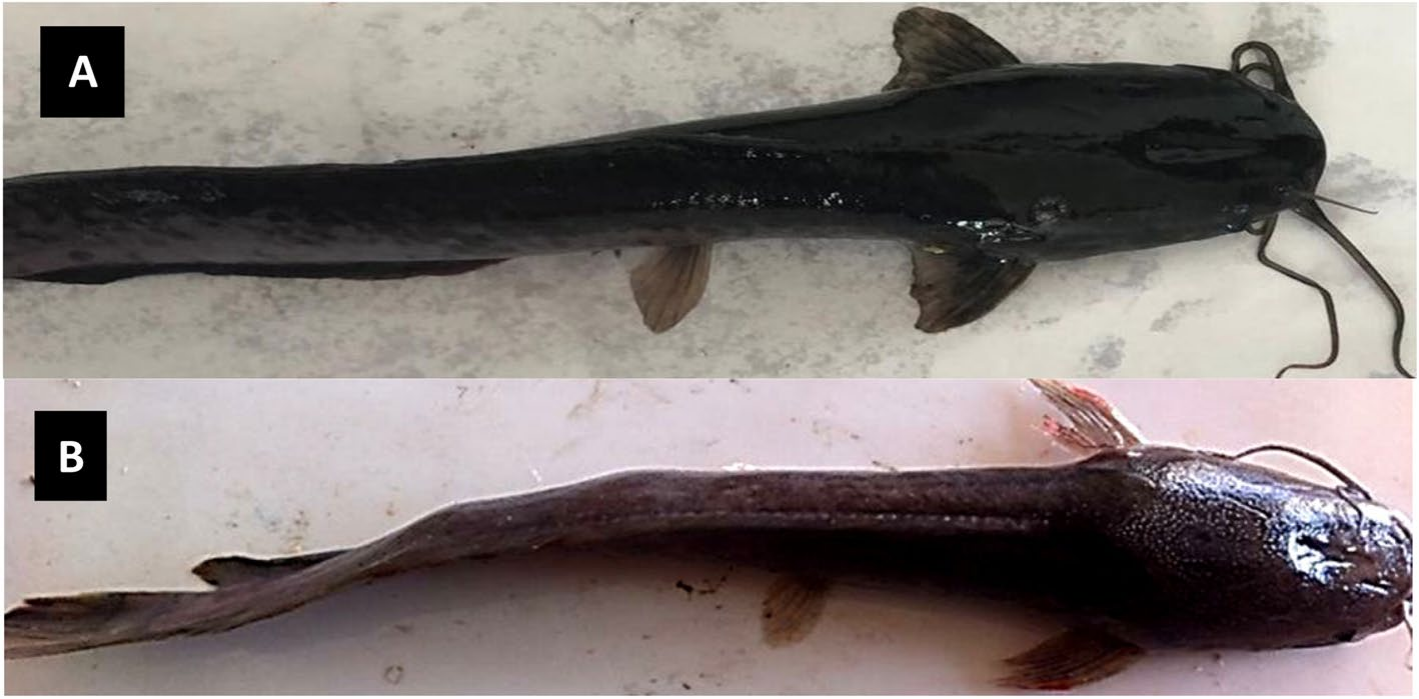
Gross examination of catfish supplemented with β-1,3-glucan fipronil and lead (**A**) versus catfish receiving fipronil and lead (**B**). Lateral deviation of the vertebral column (blue arrows); skin bleaching; and haemorrhage in the lateral fins (black arrows) were observed in the catfish group exposed to fipronil and lead nitrate, whereas those supplemented with β-1,3-glucan and combined with fipronil and lead showed no gross abnormalities

The third group of catfish (*Clarias gariepinus*) subjected to lead nitrate and fipronil had the highest mortality rate of 45%, with erections in both dorsal and pectoral fins in the dead fish (Fig. 1B). Meanwhile, the mortality rate in the fourth group, which received β1,3-glucan plus a mixture of fipronil and lead nitrate, was 5% without any abnormalities in the fins. Meanwhile, neither the untreated control group nor the − 1,3-glucan supplemented group of catfish died.

### Health status of *Clarias gariepinus*

The third group of catfish that exposed to fipronil and lead nitrate experienced a slow escape reaction and respiratory discomfort, as well as a loss of body weight, after being exposed to fipronil and lead nitrate at the aforementioned dose. Furthermore, abnormal semicircular swimming behavior and slow movement, as well as expansion of the dorso-lateral side and lateral deviation in the vertebral column, were seen. Skin abnormalities such as bleaching and pale coloration (Fig. 1B) and fish weakness predominated in the same catfish group, and fish death was linked to air bubbles and excessive mucus secretion. In the fourth group of catfish that were supplemented with β-1,3-glucan and exposed to fipronil and lead nitrate, improved physiological activities such as an active escape response to capture, no respiratory distress, no clinical signs, no change in skin color with no fin hemorrhage (Fig. 1A) and normal body weight were observed. The fish in the second group that supplemented with β-1,3-glucan, on the other hand, showed the highest physiological health activities as well as a substantial increase in fish weight among the four investigated groups. In contrast, the fish in the control group had no abnormal behavioural or clinical abnormalities and a normal body weight.

### Changes in haematological indices

At the completion of this study, various blood indices were evaluated to determine the impact of lead and fipronil pollutants on Nile catfish. According to Table 2, a combination of both pollutants (third group) significantly reduced all of the haematological parameters studied. When compared to the β-1,3-glucan non-supplemented control group, fish supplementation with β-1,3-glucan, either alone (second group) or in combination with fipronil and lead nitrate (fourth group), resulted in a significant increase in the target blood parameters.

**Table 2:** Effect of Lead, fipronil and β-1,3-glucan on some hematological and immunological parameters in *Clarias gariepinus*

Catfish groups	Hematological parameters					Immunological parameters		
	RBC (10^**12**^ L^**−1**^)	Hb (gdL^**−1**^)	HCT (%)	MCV (fL)	MCHC (gdL^**−1**^)	IL-1β (pg/ml)	IL-1β (pg/ml)	IL-1β (pg/ml)
**G1**	2.4 ± 0.032^a^	10.1 ± 0.12^a^	29.1 ± 1.3^a^	95.4 ± 9.2^a^	33.1 ± 1.3^a^	16.1 ± 0.36^a^	98.7 ± 5.3^a^	13.23 ± 0.4^a^
**G2**	2.42 ± 0.04^a^	10.2 ± 0.23^a^	29.3 ± 1.8^a^	93 ± 7.8^a^	32.9 ± 2.01^a^	18.3 ± 0.64^a^	106.1 ± 4.7^a^	15.87 ± 1.2^a^
**G3**	1.8 ± 0.1^c^	7.82 ± 0.41^c^	19.2 ± 4.1^c^	67.5 ± 9.7^c^	24.2 ± 1.6^c^	9.2 ± 0.97^c^	66.1 ± 4.56^c^	6.8 ± 0.71^c^
**G4**	2.32 ± 0.037^b^	9.97 ± 0.32^b^	28.3 ± 2.4^b^	88.3 ± 8.1^b^	32.1 ± 2.05^a^	15.7 ± 1.4^b^	92.1 ± 3.21^b^	12.9 ± .87^b^

G1: Control group

G2: β-1,3-glucan supplemented group

G3: Lead nitrate and fipronil exposed group

G4: Combined fipronil and lead, and β-1,3-glucan supplemented group

*RBC* Red blood cell, *Hb* Haemoglobin, *HCT* Hematocrit, *MCV* Mean corpuscular volume, *MCHC* Mean corpuscular haemoglobin concentration

Values are means of 10 samples ± Standard Error Mean. Column values with different superscripts are significantly different (*P* < 0.05)

### IL-1β and IL-6 and immunoglobulin M levels

The second group of β-1,3-glucan-supplemented catfish exhibited significantly higher plasma levels of IL-1β and IL-6 cytokines, as well as IgM, compared to the control catfish. In comparison to the first and second groups, the catfish in the third group that were exposed to lead and fipronil had much lower levels of IgM and IL-1 and IL-6 cytokines. In catfish of the fourth group supplemented with β-1,3-glucan and subjected to a combination of fipronil and lead nitrate, however, both IL-1 and IL-6 cytokines, as well as IgM levels, were slightly reduced, and their concentrations were similar to the control catfish concentration levels (Table 2).

### IL-1β, IL-2 and IL-6 cytokines gene expression in the tested groups

**Fig. 2:**
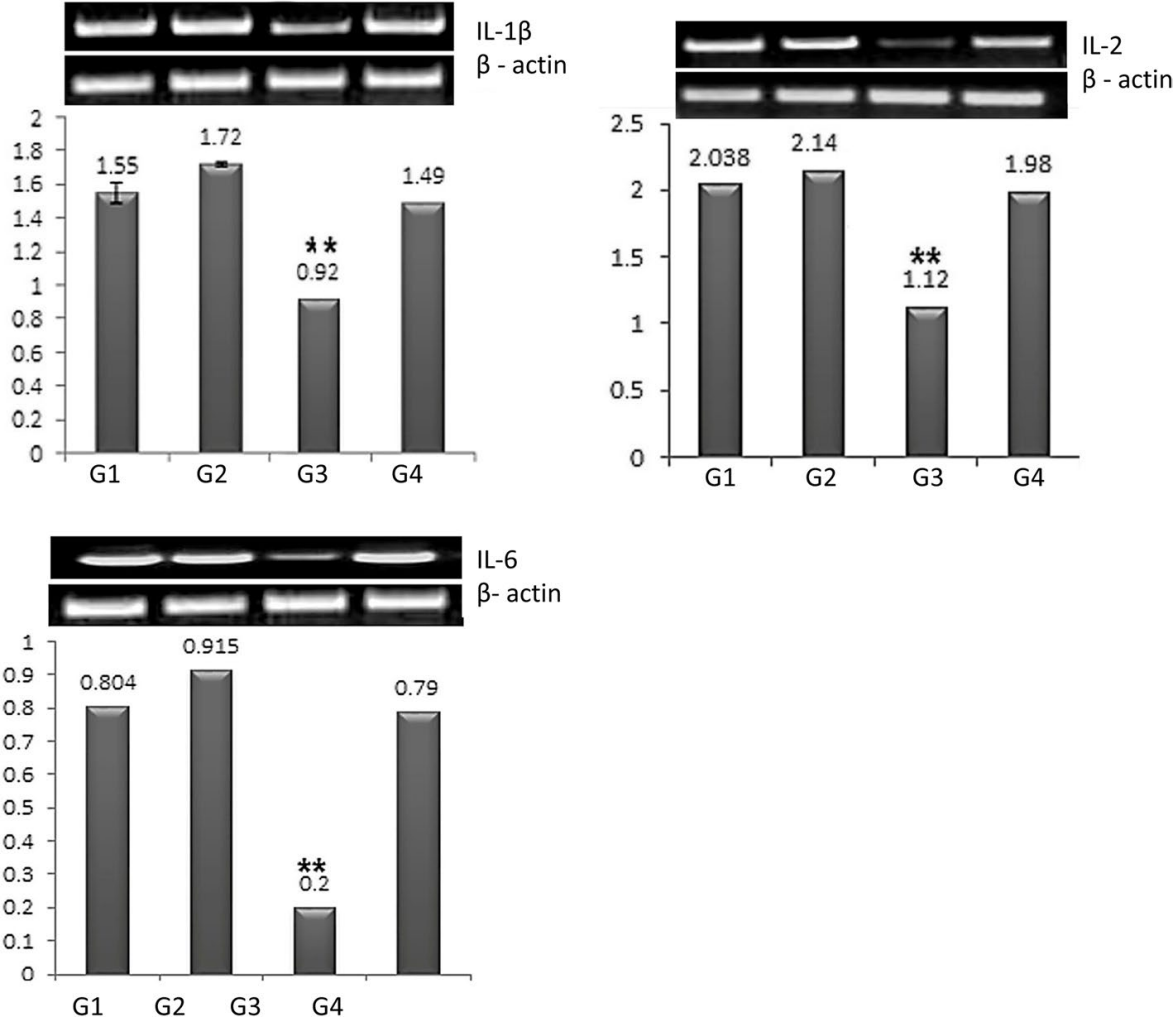
Analysis of the relative gene expression of IL-1β, IL-2, and IL-6 in four experimental *Clarias gariepinus* splenic tissues in relation to β-actin. ** denotes a significant level of significance (*P* < 0.01). G1: Control group. G2: β-1,3-glucan supplemented group. G3: Lead nitrate and fipronil exposed group. G4: Combined fipronil and lead, and β-1,3-glucan supplemented group

Semi-quantitative PCR was used to evaluate the expression of proinflammatory and inflammatory cytokine gene in splenic tissues. Figure 2 shows that the catfish supplied with β-1,3-glucan had the highest levels of IL-1, IL-2, and IL-6 expression, followed by the control non supplied group. The third group, which was exposed to fipronil and lead nitrate induced pollution at the same time, had significantly lower levels of IL-1, IL-2, and IL-6 gene expression. After β-1,3-glucan supplementation, the fourth group that received fipronil and lead nitrate had IL-1, IL-2, and IL-6 gene expression levels that were comparable to the control group.

### Histopathological findings in the intestine

**Fig. 3:**
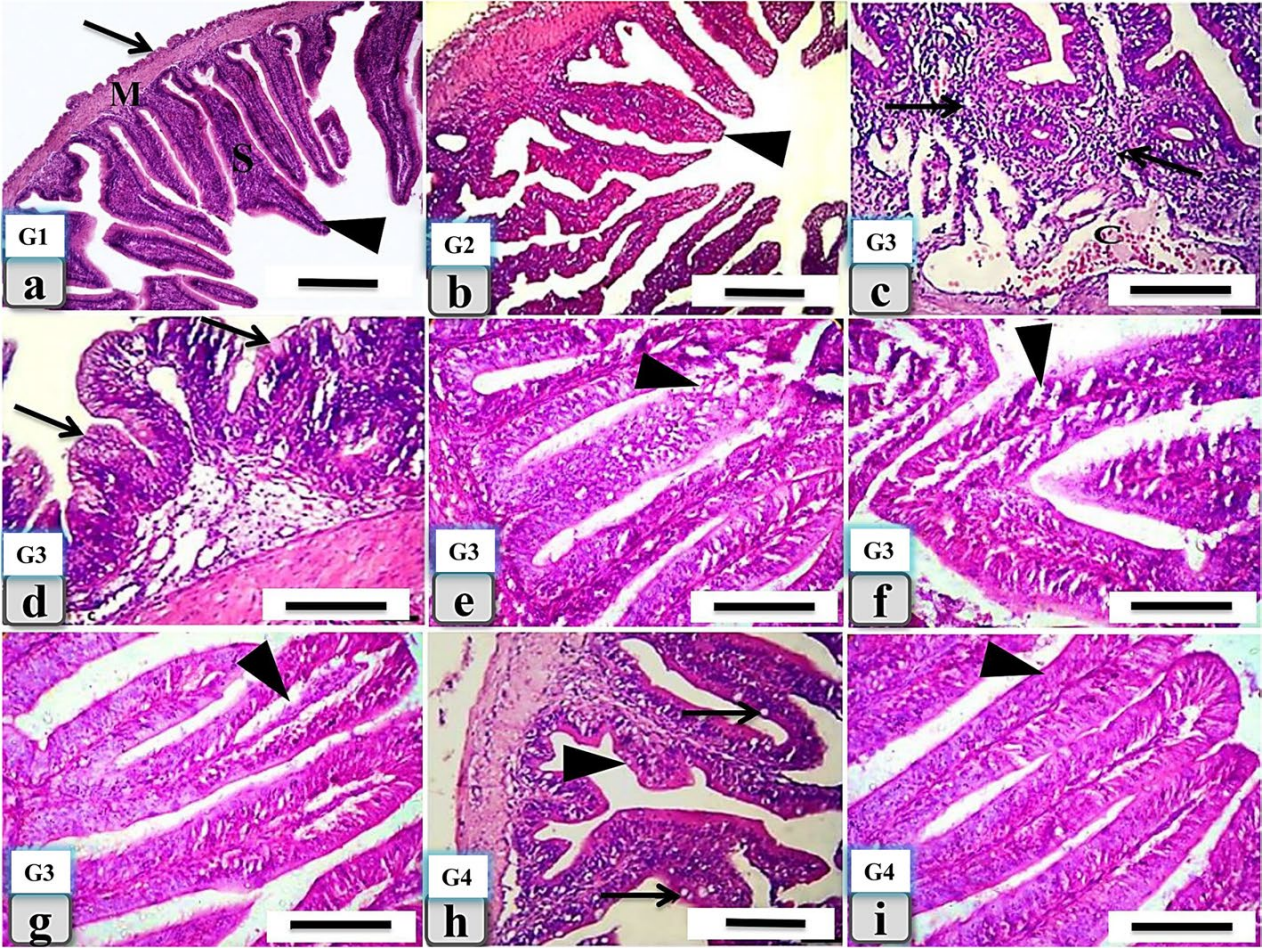
Histological changes in the intestinal tissues of the different *Clarias gariepinus* experimental groups. The control group (G1: **a**) showed a normal, intact intestinal wall; mucosa (arrowhead), submucosa (S), muscular coat (M), and serosa (arrow). The second group (G2: **b**), which was supplemented with β-1,3-glucan represented a normal, intact intestinal wall and intestinal villi with a normal lining epithelium (arrowhead). The third group (G3: **c**, **d**, **e**, **f**, and **g**) exposed to fipronil and lead revealed several histopathological views in the intestinal wall, including catarrhal enteritis with numerous lymphocytes infiltration and severe blood vessel dilatation with submucosal congestion (**c**), mucinous lining epithelial (**d**), and severe intestinal villi degeneration with focal detachment of their columnar lining epithelium [(**e**, **f**, and **g**), (arrowheads)]. The fourth group (G4: h and **i**) that received a combination of fipronil, lead, and supplemented with β-1,3-glucan showed normal, intact intestinal wall (**h**) without any abnormalities (arrowheads) and a slight increase in the goblet cell [(**h**), (arrows)] cells. Also, intact intestinal villi with normal lining epithelium [(**i**), (arrowheads)] were observed. H&E stain, Scale bar 200 μm

The intestines of the four groups were examined to determine if there were any histopathological alterations. In both the control and β-1,3-glucan supplemented groups (Fig. 3a and b), the intestine showed a normal, intact mucosa, submucosa, muscular coat, and serosa. Meanwhile, the fipronil and lead-exposed group revealed severe histopathological lesions in the intestine, including severe catarrhal enteritis with numerous lymphocyte infiltrations and severe blood vessel dilatation and congestion in the submucosa (Fig. 3c). Also, mucinous degeneration in the lining epithelium was observed (Fig. 3d). Furthermore, severe degeneration of the intestinal villi with focal detachment of their columnar lining epithelium was observed (Fig. 3e, f, and g). While the fourth group’s fish intestines showed intact walls and villi with normal lining columnar epithelium. However, mild histopathological alterations in their architecture were observed as a slight increase in the number of goblet cells with no inflammatory cell aggregations in the submucosa (Fig. 3h and i). All the previously described histopathological lesions of the intestine were recorded in Table 3.

**Table 3:** Semi –quantitative histopathological lesions score in the intestine of the of the different catfish groups

Lesion	Control group	β-1,3-glucan supplemented group	Fipronil and Lead exposed group	Combined fipronil and lead, and β-1,3-glucan supplemented group
Characteristic mucosal damage	_	_	**++**	_
Mucosal epithelial degeneration and desquamation	**_**	**_**	**++**	**_**
Degeneration in the intestinal villi	_	_	**++**	_
Goblet cell hyperplasia	**_**	**_**	**++**	**+**
Mucinous degeneration in the lining epithelium	**_**	**_**	**++**	**+**
Catarrhal enteritis	_	_	**++**	_
Lymphocytic infiltrations	**_**	**_**	**++**	**_**
Submucosal blood vessels dilatation and congestion	**_**	**_**	**++**	**_**
Proliferation and regenerative changes of the villi and its lining epithelium	**_**	**_**	_	**++**

### Histopathological findings of the spleen

**Fig. 4:**
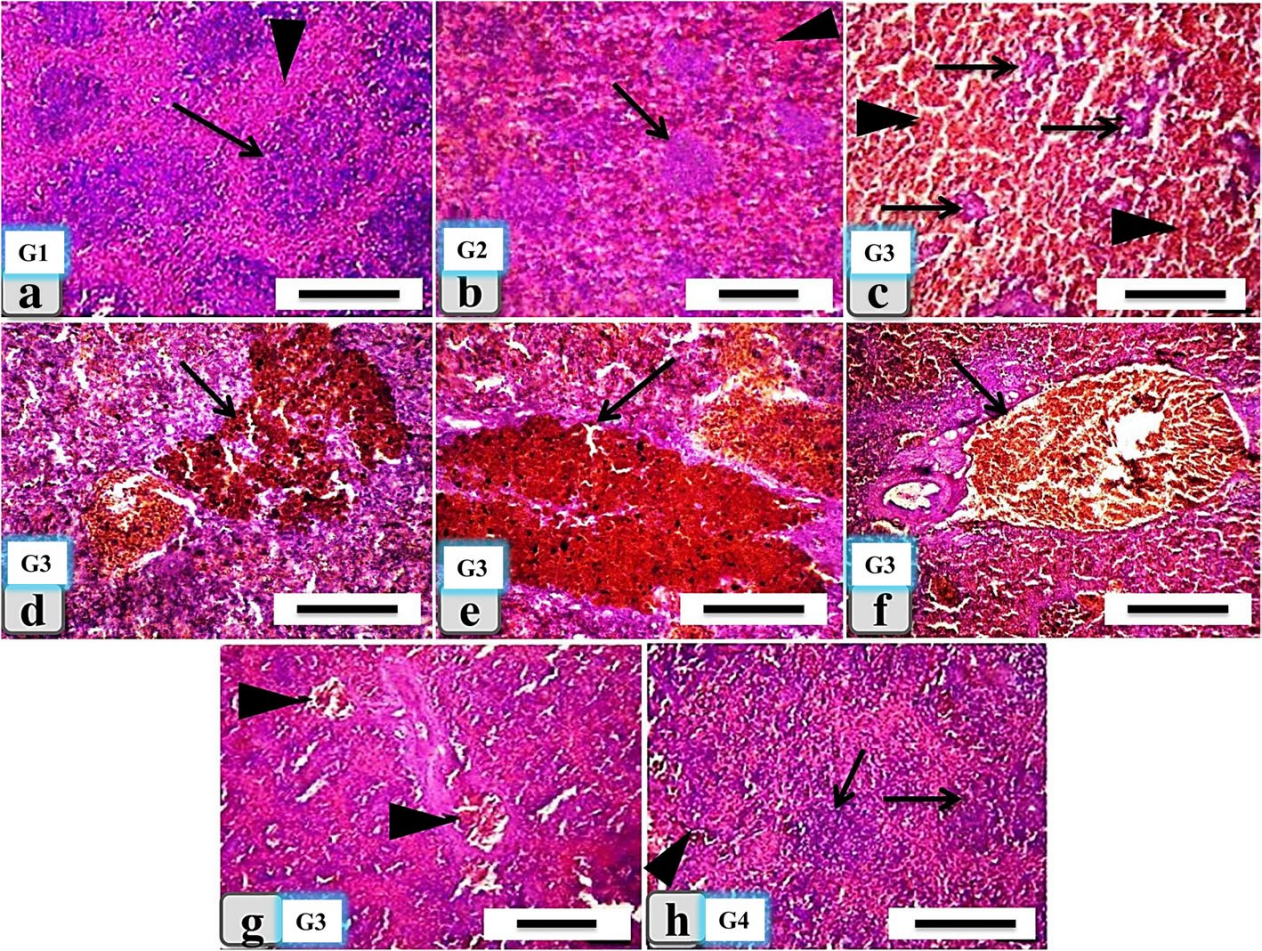
Histological changes in splenic tissues of *Clarias gariepinus* experimental groups. The control group (G1: **a**) showed normal splenic parenchyma with normal white pulp (arrow) and red pulp (arrowhead). The second group (G2: **b**) supplemented with β-1,3-glucan showed normal, intact splenic parenchyma with white pulp (arrow) and red pulp (arrowhead). The third group (G3: **c**, **d**, **e**, **f**, and **g**) that was exposed to fipronil and lead revealed severe depletion and necrosis in the lymphocytes (**c**) of white pulp (arrows), and hemorrhagic red pulp (arrowheads), severe hemosiderosis (**d**), abnormal accumulation of iron deposits (e), large golden yellow patches within the splenic parenchyma (arrow), and severe blood vessel dilatation with severe congestion (**f**) and hemorrhage (**g**) within the splenic red pulp (arrow) were all observed. The fourth group (G4: **h**) exposed to fipronil and lead combined with β-1,3-glucan showed normal splenic red and white pulp (arrows) and activated melanomacrophages (**h**) centers (arrowheads). H&E stain; scale bar = 200 μm

In both the control and β-1,3-glucan supplemented groups (Fig. 4a and b), the white and red splenic pulps were normal. Meanwhile, the fipronil and lead-treated splenic tissue showed severe depletion and necrosis in the lymphocytes of the white pulp and hemorrhagic red pulp (Fig. 4c), severe hemosiderosis, excessive accumulation of iron deposits forming large golden yellow patches of hemosiderin pigments within the splenic parenchyma (Fig. 4d and e), and severe blood vessel dilatation with severe congestion and hemorrhage within the splenic red pulp (Fig. 4f and g). In the fourth group’s fish, which received fipronil and lead and were supplemented with β-1,3-glucan the spleen revealed normal white and red pulps with activated melanomacrophage centers (Fig. 4h). All the previous histopathological lesions in the spleen were recorded in Table 4.

**Table 4:** Semi-quantitative histopathological lesions score in the spleen of the different catfish experimental groups

Lesion	Control group	β-1,3-glucan supplemented group	Fipronil and lead exposed group	Combined fipronil and lead, and β-1,3-glucan supplemented group
Depletion and necrosis in the lymphocytes of white pulp	_	_	**++**	_
Hemorrhagic red pulp	**_**	**_**	**++**	**_**
Hemosiderosis	**_**	**_**	**++**	**+**
Blood vessels dilatation with congestion in red pulp	_	_	**++**	_
Proliferation and regeneration of splenic white pulp with activated melano- macrophages centers	**_**	**_**	_	**++**

## Discussion

Fish are at the top of the food chain in most aquatic environments and are the most susceptible to the environmental pollutants. Exposure to heavy metal pollutants could impair fish’s ability to smell, affect swimming performance, reduce metabolism, damage vital organs, and could induce immunosuppression that may result in increased susceptibility to disease and mortality, in addition to transfer of toxic metabolites to fish meat and their products to the consumers. It is, therefore, important to enhance tolerance against those pollutants such as lead and fipronil that could target fish culture through waterways or the surrounding environment. Functional feeding is an emerging paradigm in the aquaculture industry that aims to develop diets of balanced nutrition supplemented with feed additives to improve both the health and disease resistance of cultured fish [51]. In this study, we looked at the effects of stress on African catfish (*Clarias gariepinus*) exposed to fipronil and lead nitrate, as well as the role of dietary β-1,3-glucan feed additives in mitigating their negative effects. The choice of Nile catfish was based on its tremendous ability to withstand environmental and aquatic stresses and its high commercial value in Egypt as one of the major economic protein sources. β-1,3-glucan was used in this study by a dose of 0.1% [43] to avoid the consequences of higher dosage side effects. A higher dosage (2%) did not reduce the induced cold-stress fish mortality, whereas a dosage of 0.5% resulted in significantly lower mortality [52]. Other studies suggested that the effect of β-1,3-glucan on stress relief is affected by dose and duration of the experiment. Β-1,3-glucan overdoses could even induce immunosuppression [38, 53, 54].

Catfish that received fipronil and lead simultaneously showed clinical signs of semi-circular swimming behavior with some dorsal swellings and vertebral column deviation, indicating poor physiological conditions and explaining the lack of escape catch response. Fipronil has been proven to disrupt the central nervous system by blocking the passage of chloride ions through the GABA receptor and glutamate-gated chloride (GluCl) channels, components of the central nervous system that result in muscle exhaustion, nervous manifestation, and respiratory distress [55, 56], which could explain the clinical signs and the high mortality rates observed in fish. A study showed that lead at concentration of 3.2 mg/L was associated with loss of balance and reduced activity could be another explanation to the semi-circular sluggish movement and the slow escape reflex seen in our study [57]. They also reported that lead induced skin bleaching and a thick layer of mucus covered the fish skin, which supports the results obtained in the current study. The overall health and physiological status data were in agreement with the earlier results [58]; and with the recent results obtained by [23] who mentioned that fipronil caused mortality in different fish species like rainbow trout, bluegill sunfish and Nile tilapia with 96 hr. LC50 of 0.246 mg L/1 and 0.083 mg L/1, respectively. These mentioned records together with the data obtained in the current study indicate the high toxicity of the fipronil and lead. It was suggested that administration of 1,3 β-glucan as dietary supplement at concentration of 0.1% [43] would ameliorate the undesirable toxic effects of fipronil and lead in catfish. Supplementation of β-1,3-glucan resulted in significant improvement of the overall fish health parameters and a lowering of mortality rates over the six-week study period. No weight loss (data not shown), abnormal movement, skin discoloration, or anatomical deviation in the vertebral column were observed in the groups that received β-1,3-glucan in the diet, confirming that it can ameliorate the detrimental effects of pollutant stress.

Blood assessment is essential for determining the physiological wellbeing and health of fish. Hematological measurements can be used to detect changes in a characteristic that exceeds its usual homeostatic limitations [59]. In the current study, the haematological indices of *catfish exposed to* lead and fipronil significantly decreased compared to the other groups of the study. These haematological alterations reported in this study can be related to direct responses to structural damage to the RBC membrane, which results in hemolysis, as well as the following requirement to rapidly create replacement blood cells to avoid anemia. This might be due to the fast oxidation of hemoglobin to methemaglobin or release of oxygen radical due to the toxic effect [60].

Different findings of some previous investigations using similar pesticides, indicating consistencies in haematological responses. There was significant decrease in erythrocytes count in fipronil and lead exposed groups compared with control (Table 2). Previously, [23, 61] reported that fipronil at very low concentration 0.0002 mg/L (0.2 μg/L) causes lower platelets counts and Hb concentrations in *Cyprinus carpio* L. [62]; erythrocyte injury in silver catfish, *Rhamdia quelen* as a result of detrimental effect of fipronil on erythrocytes synthesis. However, these results were differed from those reported by [63, 64] who mentioned that neither hemoglobin concentration nor total erythrocytic count was affected when buffalo calves were exposed to fipronil at dose level (0.5 mg/kg body weight per day). This disagreement most probably will be due to the different species.

The histopathological findings in the intestines of both the control and β-1,3-glucan supplemented groups had normal, intact intestinal villi with normal lining columnar cells and an intact intestinal wall, as well as normal mucosa, submucosa, muscular coat, and serosa and were similar to the results of [65]. Meanwhile, those catfish exposed to the fipronil and lead nitrate had severe histopathological lesions in the intestine. It was described previously [23] that the intestine of fish subjected to 0.014 mg/l of fipronil for 4 days showed severe necrosis in the intestinal villi that were infiltrated with lymphocytes and macrophages, desquamated epithelium, leukocytes, and erythrocytes, with severe mucinous degeneration. Recent study reported that *Oreochromis niloticus* group that received a low dose of fipronil showed features of catarrhal enteritis with desquamation in the epithelial cell and inflammatory cell infiltration. Meanwhile, those received a high dose of fipronil showed severe necrotic enteritis, sluffing of the epithelial cells, and blunting of the villi [28].

Furthermore, the splenic tissue in the fipronil and lead-exposed group showed severe depletion and necrosis of white pulp and hemorrhagic areas in red pulp, severe hemosiderosis, severe blood vessel dilatation, and severe congestion. A recent study [28] on *Oreochromis niloticus,* clarified that the group received a low dose of fipronil showed congestion of the red pulp and lymphoid depletion compared to a significant dose-dependent lymphoid depletion in the group that received a high dose of fipronil, as well as severe necrosis of lymphoid and melanomacropahges cells. In the current study, the intestine and spleen of fourth group’s catfish that received combined β-1,3-glucan with fipronil and lead nitrate intestine and spleen showed mild histopathological alterations as mentioned in the results. In addition, the spleen revealed normal white and red pulps with activated melanophage centers. These findings suggested that β-glucan is a promising dietary supplement that can improve catfish and induce histopathological changes and act as a fipronil and lead pollutant neutralizing agent in catfish culture.

Our data showed that supplementing catfish diet with β-1,3-glucan significantly increased IL-1β and IL-6 cytokines, as well as IgM levels, confirming previous findings in other fish species and implying the immunostimulatory activity of β-glucans [27, 39, 66, 67]. These cytokines stimulate the formation of new white blood cells, providing immunity to β-glucan binding receptors found in all vertebrates, from fish to humans [68]. β-glucan supplementation has been shown to modulate the immune response and increase resistance to microbial infection in Nile tilapia [69]. The production of these cytokines may be due to the binding of macrophage lectin receptors to β-1,3-glucan that result in improvement of the different immune functions such as phagocytosis, the release of certain cytokines such as IL-1, IL-6, GM-CSF, and interferons, and antigen processing. The effects of dietary β-glucan was investigated in silver catfish (*Rhamdia quelen*) and Nile tilapia [65, 70]. They discovered that supplementing with β-glucan has a significant effect on growth performance, blood cells, serum bacterial agglutination, immune-related genes, and serum myeloperoxidase activity. In addition, fish of the β-glucan treatment were challenged with *A. hydrophila* and showed fewer bacteria in blood and presented a significantly higher survival rate compared to the control group [65] confirming its immunostimulatory activity.

In-vitro studies indicated that β-glucan elicited pro-inflammatory cytokines by activated macrophages [71]. However, feeding β-glucan to fish does not improves total or specific immunoglobulin production, as reported in Nile tilapia (*Oreochromis niloticus*) [72], common carp [73], rainbow trout [74] and gilthead sea bream, [75] which contradicts to our findings. Fish species, feeding regimens, or nature of β-glucan and its dosages could all explain this variation. The increased levels of catfish major cytokines and the main protective and neutralizing antibody, IgM, reported in this study could explain the improved resistance to various aquatic pathogens in different fish species observed in other studies [51, 76, 77]. Furthermore, feeding β-glucan increased the expression of immune-related cytokine genes in catfish, as evidenced by higher serum levels.

## Conclusion

In conclusion, the aquaculture industry is growing rapidly, and its protection against pollutants is required to make growth sustainable and increase yield and quality, so feed additives should be used. β-Glucan is a new prebiotic in aquaculture that requires much more research to understand its roles in different species of farmed fish. According to our research highlighted that adding β-glucan to the diet can improve health, neutralize histological changes, and boost immunity in catfish cultures exposed to fipronil and lead pollutants. Taken together, we would strongly advise using β-glucan in farmed fish diets whenever there is sufficient evidence for its benefit, with the goal of improving overall health. More research should be done to optimize the dosage and improve the quality of β-1,3-glucan in different cultured species. However, chronic bioassays should be performed to ensure the fish exposure to those toxic pollutants’ safety.

## Supplementary Information

**Additional file 1: Supplementary data.** Semi-quantitative PCR of IL-1β, IL-2, and IL-6 cytokines in the experimental catfish groups.

## Data Availability

The datasets used and/or analyzed during the current study available from the corresponding author on reasonable request.
