# Supplementary material for: β-1,3-glucan improved the health and immunity of juvenile African catfish (Clarias gariepinus) and neutralized the histological changes caused by lead and fipronil pollutants
Source: BMC Vet Res. 2023 Feb 11;19:45. doi: 10.1186/s12917-023-03585-5 (PMC9921358; doi:10.1186/s12917-023-03585-5)
Supplement: Supplementary file 1 — Additional file 1: Supplementary data. Semi-quantitative PCR of IL-1β, IL-2, and IL-6 cytokines in the experimental catfish groups. [file 12917_2023_3585_MOESM1_ESM.pptx]

## Slide 1
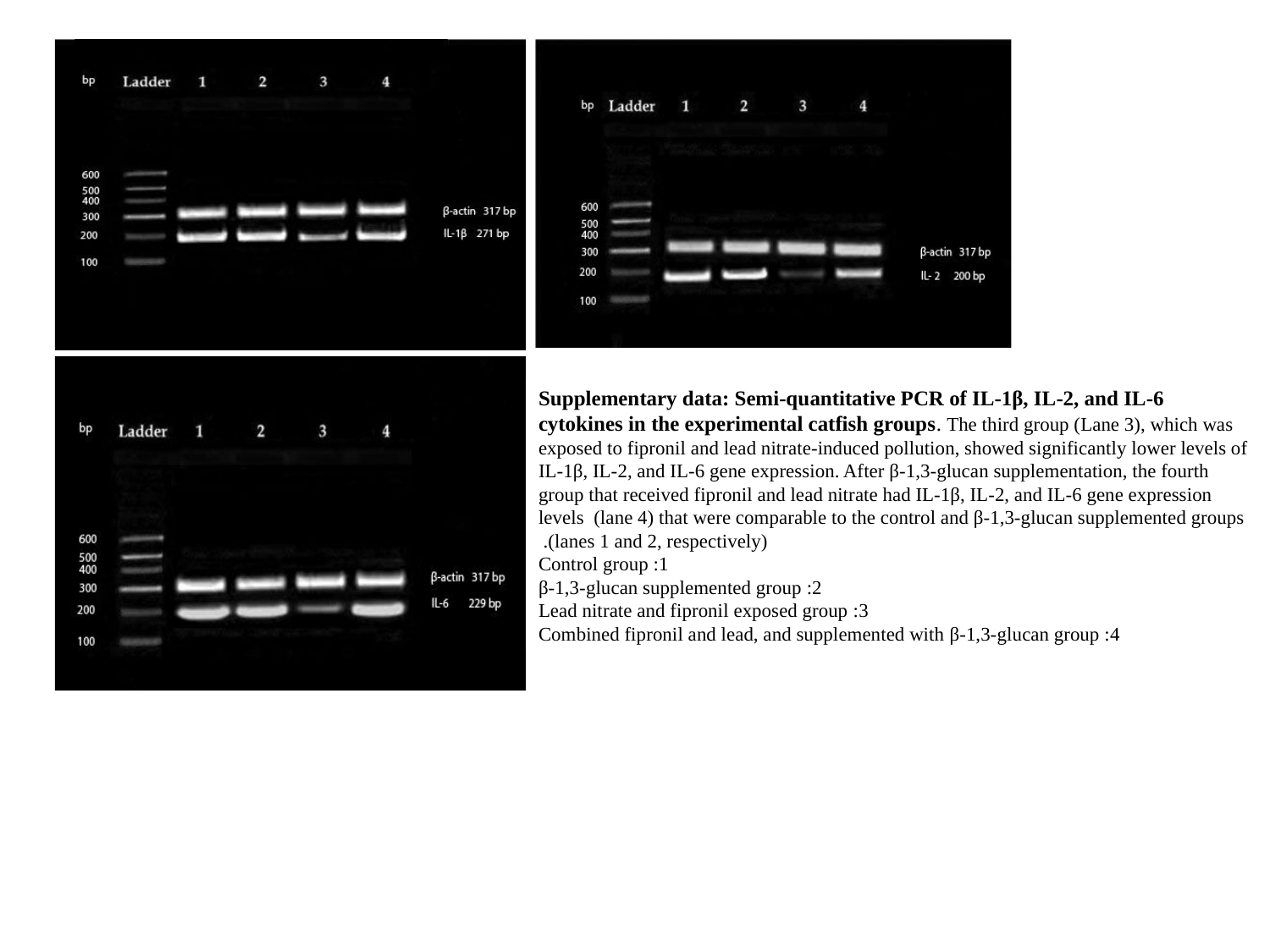

Supplementary data: Semi-quantitative PCR of IL-1β, IL-2, and IL-6 cytokines in the experimental catfish groups. The third group (Lane 3), which was exposed to fipronil and lead nitrate-induced pollution, showed significantly lower levels of IL-1β, IL-2, and IL-6 gene expression. After β-1,3-glucan supplementation, the fourth group that received fipronil and lead nitrate had IL-1β, IL-2, and IL-6 gene expression levels  (lane 4) that were comparable to the control and β-1,3-glucan supplemented groups (lanes 1 and 2, respectively).
1: Control group
2: β-1,3-glucan supplemented group
3: Lead nitrate and fipronil exposed group
4: Combined fipronil and lead, and supplemented with β-1,3-glucan group
